# From Qualitative Localisation to Quantitative Verification: Integrating Active IR Thermography and Laser Scanning in Wind Turbine Blade Inspection

**DOI:** 10.3390/ma19061107

**Published:** 2026-03-12

**Authors:** Adam Stawiarski

**Affiliations:** Department of Machine Design and Composite Structures, Faculty of Mechanical Engineering, Cracow University of Technology, Al. Jana Pawła II 37, 31-864 Krakow, Poland; adam.stawiarski@pk.edu.pl

**Keywords:** infrared active thermography, composite blades, 3D laser scanning, reverse engineering, geometry-aware NDT, quality assessment, coupled damage detection systems

## Abstract

A coupled non-destructive testing (NDT) workflow is proposed that integrates active infrared thermography (IRT) with laser-scanning-based reverse engineering (RE) to increase the reliability of detecting and interpreting damage in composite wind turbine blades across laboratory specimens and real components. IRT provides rapid, image-based qualitative localisation of potential anomalies, while 3D scan analysis supplies quantitative, geometry-aware verification and measurement of defect magnitude, reducing both false positives (design-related thermal signatures) and false negatives (weak thermal contrast). On polystyrene-filled profiles, IRT alone produced thermal anomalies unrelated to delamination; co-registered scan maps identified or ruled out local indentation, correctly attributing heat-flow patterns to internal design rather than damage. Outcome: the fused method disambiguates thermal indications and quantifies defect magnitude. On a vertical-axis wind turbine (VAWT) blade, the integration distinguished genuine geometric change from architectural effects under unknown internal structure and without CAD/reference scans, preventing false calls. For three horizontal-axis wind turbine (HAWT) blades, fleet-level scan comparison detected a significant tip deviation despite no clear local IRT anomalies, demonstrating complementary roles: scan = global quantitative homogeneity; and IRT = local qualitative verification. These findings operationalise thermal–geometric cross-validation and outline a path toward UAV-enabled inspections combining passive IRT and laser scanning for hard-to-access structures under real environmental conditions.

## 1. Introduction

The rapid expansion of composite applications in modern wind turbine blades has highlighted the need for advanced non-destructive testing (NDT) methods that ensure structural integrity throughout the entire life cycle of turbine components. Composite laminates and sandwich structures exhibit multiple interacting failure mechanisms, including delaminations, fibre waviness, core–skin disbonds, matrix cracking, and progressive fatigue damage. These mechanisms arise both from manufacturing defects and service-induced environmental loading, such as strong winds, icing, sand erosion, humidity, and lightning strikes [1,2,3,4]. A comprehensive review of failure mechanisms in wind turbine blades indicates that the complexity of multilayer composite architecture and variability in loading paths challenge conventional design models and inspection strategies [5,6,7].

Non-destructive testing techniques have evolved alongside the increasing demand for reliable assessment of large composite blades. Ultrasonic testing, both in laboratory settings and embedded within structural health monitoring (SHM) systems, remains the most established method for internal defect detection [8,9,10]. Acoustic emission (AE) and guided wave propagation approaches offer additional sensitivity to damage initiation and evolution, particularly under cyclic and fatigue loading [11,12,13,14,15]. Despite their high sensitivity, ultrasonic-based methods require physical contact, coupling agents, and controlled access conditions, which limit their applicability for fast in-field inspections of long, curved blades. Vision- and image-based inspection systems, enhanced by photogrammetry, unmanned aerial vehicle (UAV) platforms and automated image processing, have gained prominence due to improvements in imaging sensors and robotics integration [16,17].

Infrared thermography (IRT) is widely recognised as one of the most efficient full-field NDT methods for composite structures, especially in the aerospace and wind energy sectors [18,19,20,21]. Active thermography, relying on external excitation sources such as halogen lamps, flash lamps, laser-based heating, or ultrasonic stimulation, enables rapid detection of subsurface defects in GFRP and CFRP components. Field applications of IRT on wind turbine blades, including large-scale on-site campaigns and analyses of environmental influences, have shown both its strengths and limitations. Factors such as solar radiation, wind speed, surface contamination, and blade orientation can substantially affect thermal contrast and the interpretation of thermograms [22,23,24]. Recent research emphasises that careful scheduling of inspections, coupled with predictive modelling of thermal gradients, significantly enhances defect detectability under operational conditions [25,26,27].

The integration of unmanned aerial vehicles has transformed wind turbine blade inspection by enabling high-resolution optical and thermographic imaging, without the need for rope access or turbine shutdown. UAV-mounted thermal cameras, together with automated path planning and machine learning-assisted damage detection, support real-time assessment of erosion, cracking, and delamination [28,29]. However, the interpretation of thermal data remains challenging when acquired at varying distances, angles, and environmental conditions. Consequently, there is a growing trend towards multi-modal NDT and data fusion approaches that combine the strengths of different techniques. Examples include fusing pulsed thermography with phased-array ultrasonics, combining IRT with digital shearography, and integrating thermographic imaging within 3D scanning or structured-light measurement frameworks to produce geometry-aware damage maps [30,31,32].

In parallel with the development of conventional SHM techniques such as ultrasonic testing, acoustic emission and guided waves, recent advances in carbon-based sensing technologies have gained considerable attention. In particular, carbon-nanotube (CNT)-enhanced sensors and CNT-integrated composite laminates demonstrate high sensitivity to strain and damage initiation, offering real-time monitoring capabilities and early-warning functionalities that complement externally mounted NDT systems. Their piezoresistive behaviour enables distributed strain mapping and damage detection under complex loading conditions, providing an additional pathway for the SHM of large-scale composite structures, including wind turbine blades [33]. Incorporating such embedded or surface-applied CNT networks into inspection frameworks has been shown to improve diagnostic reliability, particularly in detecting micro-cracking, interfacial debonding and progressive fatigue damage.

Although the combination of active thermography and high-fidelity laser scanning offers significant advantages, several limitations must be acknowledged. First, accurate co-registration of thermal data on three-dimensional geometries becomes increasingly challenging for highly curved or free-form surfaces, which are common in modern blade designs generated through aerodynamic optimisation algorithms. Surface complexity may introduce viewpoint-dependent distortions, occlusions, or registration errors that affect defect localisation. Second, thermal response and emissivity behaviour depend strongly on the material system, laminate architecture, and surface coating, which may reduce the reliability of geometry-normalised contrast metrics if not properly compensated. These aspects highlight the need for further developments in adaptive geometry processing, emissivity modelling and automated calibration routines for broader applicability of the proposed framework.

Despite these advancements, a significant methodological gap persists regarding the systematic combination of active infrared thermography with reverse engineering (RE) workflows—particularly laser scanning and structured-light 3D reconstruction—when inspecting full-scale wind turbine blades. The ability to co-register thermal anomalies onto accurate three-dimensional blade geometries is crucial for distinguishing true subsurface flaws from geometry-induced artefacts, such as curvature-driven temperature variations or differences in emissivity [30,31,32]. Hybrid approaches merging IRT with 3D scanning have shown promising results in aerospace composite structures, yet equivalent procedures tailored to wind turbine blades, including environmental compensation, are not fully developed [30,32].

This article proposes a unified methodology that integrates active infrared thermography with high-fidelity laser scanning within a reverse-engineering framework to improve defect detection, localisation, and characterisation in composite wind turbine blades. The contributions include: (i) a workflow for precise co-registration of thermal images onto 3D blade meshes; (ii) geometry-based normalisation routines enhancing the reliability of thermal contrast; (iii) a comparative performance evaluation of standalone and integrated methods; and (iv) validation on material specimens and real-scale blades with vertical and horizontal axes of rotation. The resulting methodology addresses current limitations of single-modality inspections and forms a foundation for future extensions towards fully autonomous UAV-based multi-modal inspection systems. In this article, laser-scanning-based reverse engineering is combined with active infrared thermography to exploit the complementary advantages of both methods. Coupling the techniques reduces the likelihood that a significant fault remains undetected by either method alone, thereby improving safety and reliability in the inspection of composite structures for wind-turbine applications.

## 2. Materials and Methods

Composite materials have been used in the construction of wind turbine blades for many years and are exposed to numerous types of damage arising from operational loading, environmental exposure, or imperfections introduced during manufacturing. The wide variety of possible failure modes complicates the theoretical assessment of structural durability, while the presence of diverse protective surface layers additionally affects the sensitivity and reliability of non-destructive testing (NDT) and condition-monitoring systems. To investigate the influence of surface finish and internal structure on the performance of active infrared thermography and laser-scanner-based reverse engineering, two categories of test objects were considered in this study.

The first category comprised full-scale wind turbine blades and blade profiles for which no detailed information on internal structure, material configuration, or manufacturing process was available. Such components realistically reflect conditions encountered in engineering practice, where inspection procedures must operate without prior knowledge of laminate architecture, infill materials, or production techniques. Their inclusion allowed an assessment of the robustness of the coupled NDT approach under conditions of structural uncertainty.

The second category consisted of laboratory-manufactured composite elements produced specifically to provide controlled reference conditions for method evaluation. These specimens were fabricated using glass fibre fabric in a twill weave (thickness approx. 0.3 mm) impregnated with epoxy resin Epidian 601 (Sarzyna Chemical Sp. z o.o., Nowa Sarzyna, Poland), following the process recommended by the material manufacturer. Each laminate was constructed from two plies oriented at 0/90°, where the 0° direction was aligned with the blade’s leading edge. Fabrication was carried out using the vacuum bag technique with an applied vacuum level of approximately 0.8 bar, ensuring uniform compaction of the laminate. The curing process proceeded under ambient temperature conditions, reflecting typical low-temperature composite manufacturing routes.

**Figure 1:**
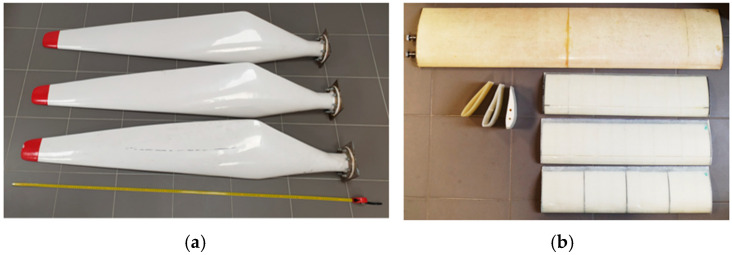
(**a**) Set of blades for wind turbines with horizontal axes of rotation (HAWT), as well as (**b**) profiles and blades used in turbines with vertical axes of rotation (VAWT).

These laboratory-fabricated components, together with industrially produced blades of unknown manufacturing history, enabled verification of the effectiveness of the coupled thermography–laser scanning methodology across different structural scales and material configurations. Figure 1 illustrates the range of examined geometries, including horizontal-axis (HAWT) blades and vertical-axis (VAWT) blade profiles, with the latter fabricated using the vacuum bag method with glass-epoxy laminates or by incorporating polystyrene infill to replicate selected industrial designs.

### 2.1. Non-Destructive Damage Detection by Active Infrared Thermography

**Figure 2:**
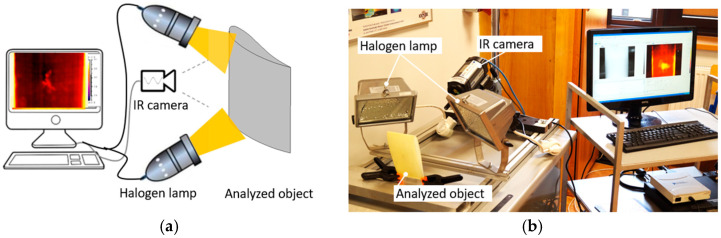
(**a**) Schematic of the reflection thermographic measurement and (**b**) photograph of the experimental set-up.

Infrared thermography is a damage detection method that records the surface temperature distribution of the inspected object and its response to a controlled thermal impulse. The thermograms, recorded at a given frequency, are processed to visualise regions where heat-flow behaviour deviates. The effectiveness of the use of infrared thermography in the context of damage detection depends, among other things, on the thickness and type of material and the adaptation of the measurement technique. In this work, reflection measurement technique was employed, in which the source of the thermal pulse and the thermal imaging camera were positioned on the same side of the test object (Figure 2). In the thermographic analyses, the FLIR A325 (FLIR Systems, Wilsonville, OR, USA) infrared (IR) camera has been used to record the surface temperature profile. The IR camera was used with the following parameters: resolution 320 × 240, spectral range 7.5–13 μm, frame rate 60 Hz, and temperature range −20 to 350 °C. The data analysis was carried out with the help of IrNDT v1.7.2 software and ThermaCAM Researcher Pro 2.10 software.

While infrared thermography can be applied as a stand-alone technique, reliable use requires experience in tailoring the measurement protocol to the material under test and the anticipated damage type. Moreover, under in-service or outdoor conditions, changing environmental factors (e.g., solar loading, wind, ambient gradients, surface contamination, emissivity) may affect both passive and active thermography, complicating the interpretation of results.

### 2.2. Laser Scanning for Damage Detection and Quality Assessment

**Figure 3:**
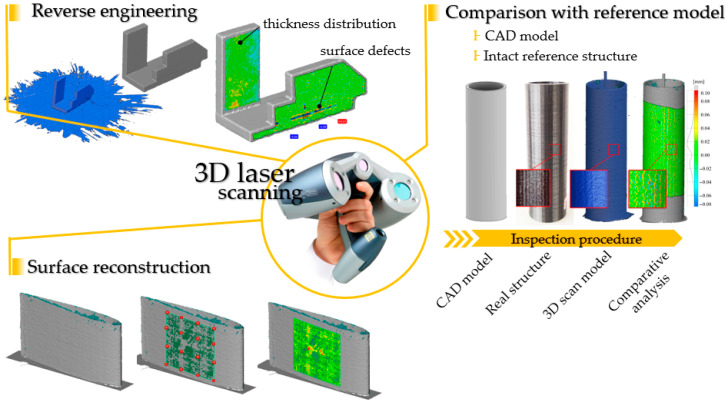
3D scanning workflow for quality control and damage detection.

Laser scanning is widely used in reverse engineering to digitise real objects. Advances in sensor technology and computing performance have enabled non-contact, high-resolution scanning to support the quality control of structural components and damage detection. In this study, manufactured structures were scanned using a Creaform REVscan handheld, self-positioning 3D laser scanner (Amtek Company, Arnold, MD, USA) and evaluated with ZEISS Inspect 2025 software. The REVscan acquires 18,000 points per second with an accuracy of up to 50 μm. A diagram of the use of a laser scanner in quality control and defect detection is shown in Figure 3. The primary use of the scanner is to digitise real objects without precise information about the source component.

Reverse-engineering techniques convert point-cloud data (including point filtering, edge extraction and surface reconstruction) into quantitative information, such as thickness distribution or surface-quality metrics. A complementary route involves comparing the scanned object against a reference. This approach is commonly used for manufacturing process assessment and periodic quality control. The reference may be an ideal CAD model of the part or a scan of an undamaged structure, examined at defined intervals. In engineering practice, it is often the case that only a single digitised state of the object is available, with no baseline (initial or undamaged) information. In such cases, one can reconstruct basic geometric entities (edge, planar or polynomial surfaces, solids) from selected points on the scan and treat deviations from the reconstructed ideal as potential damage. All of the above workflows—whether absolute (baseline/reference-based) or relative (reconstruction-based)—are subject to uncertainty, which may lead to ambiguous or inaccurate results. Outcome quality depends on factors such as scanner accuracy (point-cloud registration precision in a three-dimensional space), the effectiveness of matching algorithms between the reference and digitised object, and the robustness of surface-quality descriptors (numerical measures of deviation relative to the reference component).

## 3. Results

### 3.1. Damage Detection in Wind Turbine Blade Profiles

**Figure 4:**
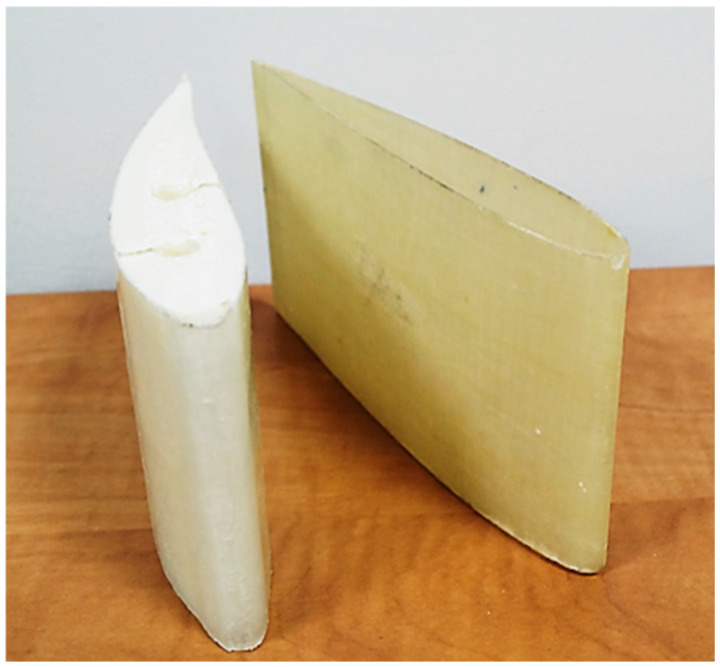
The vertical-axis wind turbine blade profiles made of GFRP composites and GFRP composites filled by polystyrene.

The main objective of this study was to evaluate the effectiveness of combining active infrared thermography with 3D laser scanning for detecting damage in different types of composite wind turbine blade structures. To enable small-scale verification of the proposed dual-method inspection approach, simplified wing-profile models of a vertical-axis wind turbine were prepared using glass-fibre-reinforced polymer (GFRP) composites (Figure 4). One profile consisted solely of a glass–epoxy laminate, whereas the second incorporated a polystyrene core, reflecting common material configurations encountered in full-scale blades. Details of the manufacturing process for these laboratory-made models are provided in the dedicated Materials and Methods subsection and are not repeated here. Damage was introduced in both profiles through a single impact delivered by a spherical steel impactor, ensuring a repeatable and clearly identifiable defect. The prepared models thus enabled a focused assessment of how the integrated thermography and scanning methodology performs under simplified yet representative structural conditions relevant to wind turbine blade inspection.

**Figure 5:**
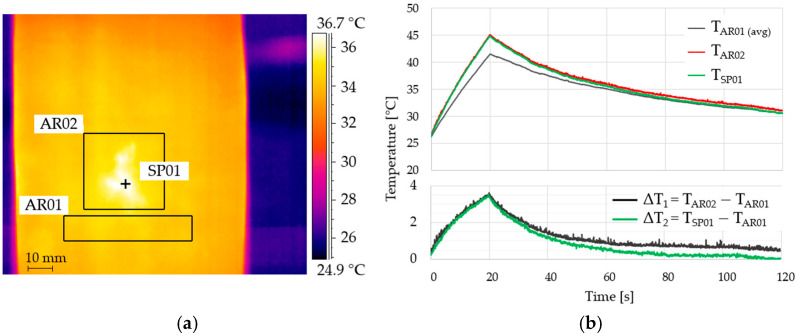
Results of the thermographic analysis of the composite profile without infill: (**a**) surface temperature distribution at t = 10 s (heating); (**b**) temperature and thermal contrast vs. time for defective and reference areas. Notation: AR01—reference area (sound material) used to compute the average surface temperature (T_AR01_); AR02—suspected defect area used to track the maximum temperature within the region (T_AR02_); SP01—single pixel/point located at the centre of the suspected defect; ΔT—temperature difference between a defective location (AR02 or SP01) and the reference (AR01), used as a scalar indicator of anomaly magnitude. ΔT_1_ = T_AR02_ − T_AR01_, ΔT_2_ = T_SP01_ − T_AR01_.

The active infrared thermography test involved generating a 20 s heat pulse using two 400 W halogen lamps (Figure 2). The thermal imaging camera recorded thermograms at 15 Hz during heating and cooling of the sample to ambient temperature. The total time for collecting measurement data was 120 s. IR-NDT v1.7.2 software was used to process recorded thermograms from the entire analysis run, highlighting defects based on differences in temperature waveforms at various points within the structure under study (Figure 5). The resulting dataset supports both the visual inspection of thermograms and multi-point, time-series temperature analysis against a reference response.

The temperature field was recorded throughout heating and subsequent cooling. To obtain quantitative indicators for damage detection, three signals were analysed: (i) the average temperature over the reference area (AR01), (ii) the maximum temperature within the suspected region (AR02), and (iii) the temperature at a single point located at the defect centre (SP01). The time histories show consistently higher temperatures at AR02/SP01 than at AR01 during the heating stage, indicating reduced through-thickness heat flow compatible with a subsurface imperfection. The corresponding contrasts ΔT_1_ = T_AR02_ − T_AR01_ and ΔT_2_ = T_SP01_ − T_AR01_ peaked at approximately 4 °C, providing a clear, scalar measure of anomaly magnitude. During the early cooling stage, point-wise measurements (SP01) can converge faster to the reference due to local emissivity/geometry and noise sensitivity; therefore, for robust detection we prioritise area-based metrics (AR02 vs. AR01) and peak-contrast values obtained during heating. This choice is consistent with practical IRT deployments, in which area statistics improve signal-to-noise ratio and reduce the need for a priori knowledge of the exact defect location.

It is worth noting the difference in the analysis of the maximum temperature value measured at the surface covering the defect (AR02) and at the defect point (SP01) during cooling. During the initial phase of temperature reduction, the temperature measured at the SP01 point differs significantly from the reference temperature of the undamaged area. However, relatively quickly over the course of the analysis, the temperature at point SP01 equalled the average temperature of area AR01. Thermal numerical indicators are used in damage detection algorithms. The results suggest that, in terms of the effectiveness of damage detection systems, the phase during which the structure’s response to a thermal impulse is recorded is more important than the cooling-down phase. During the heating phase, the temperature measured at the point and in the potentially damaged area clearly shows the temperature difference. However, during the cooling phase, interpreting the results based solely on the temperature at the point can lead to erroneous or ambiguous damage detection results. Moreover, in terms of the practical application of active infrared thermography, it is more practical to record the maximum temperature in an area for both damage detection and structural condition monitoring systems. In structural monitoring systems, indicating the temperature recording point requires prior knowledge of the location of potential damage.

**Figure 6:**
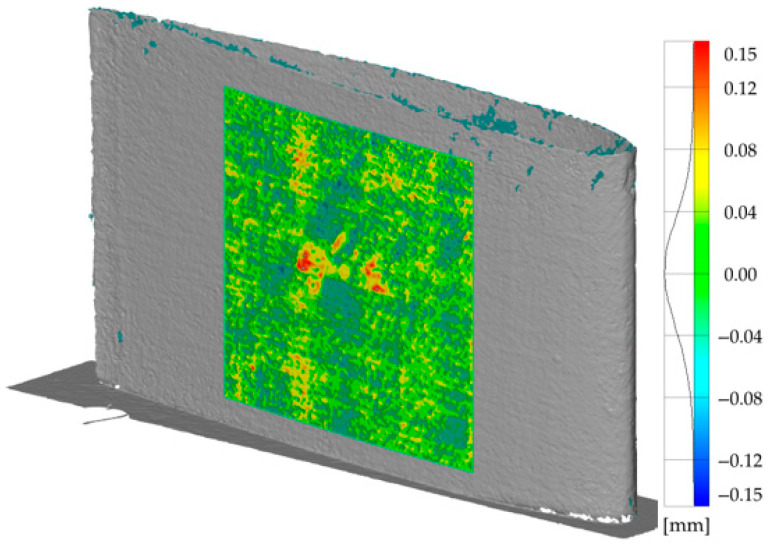
Results of the damage detection based on the 3D scan analysis.

The analysed profile of the unfilled wing was scanned at the maximum possible resolution, which facilitated the reconstruction of the geometry on the basis of an excess of 2 million points collected on the surface of the studied object. In the selected square area, a polynomial surface that best matched the recorded points was created based on the collected points. This method allows filtering of the collected data and eliminating outlier points. An analogous method is employed in cartography, whereby data is filtered from aerial scans of terrain topography for the identification of objects that do not belong to the scanned land surface (e.g., buildings). The matched polynomial surface facilitates the determination of a reference object, upon which all collected measurement points can subsequently be compared. This is particularly advantageous in circumstances where a reference CAD model is not available for comparison with the measurement data. As illustrated in Figure 6, the outcomes of the comparative analysis of the collected measurement data with the fitted polynomial surface are presented.

The scale indicates the deviation of individual measurement points from the reference surface. The histogram of values next to the scale demonstrates a very good match between the measurement points and the generated reference surface. The areas highlighted in red indicate the detection of potential damage, as indicated by a relatively high level of deviation in the position of the measurement points relative to the reference surface. In the case presented here, both methods of damage detection allowed unambiguous identification of the damaged area despite the absence of a reference object (a CAD model for laser scanning and the thermal response of an undamaged reference object for thermography). In terms of information content, active IRT (with a vision pipeline over thermograms) provides qualitative detection of a potential defect and supports localization on the thermal image (through defined regions/points). 3D scan analysis offers quantitative assessment, capturing not only the presence of an anomaly but also its magnitude via geometric deviation maps. Therefore, the combined use of IRT and scanning yields a more complete and precise non-destructive evaluation than either method alone by cross-validating thermal and geometric indicators and reducing misinterpretation risks.

**Figure 7:**
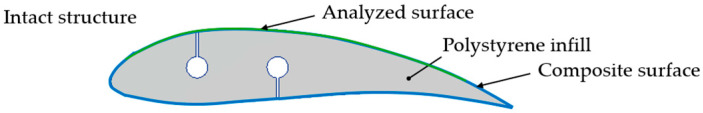
Cross-section of the polystyrene-filled blade profile indicating the internal design and the location of the analysed region.

An analogous analysis was made of a blade section filled with extruded polystyrene. Vertical-axis wind turbine blades use a different infill design, often incorporating elements to stiffen the profile. In the object under study, holes were prepared for aluminium stiffening rods made by burnishing with a resistance wire (Figure 7). The insertion point of the resistance wire leaves a gap that can be detected by damage detection systems.

**Figure 8:**
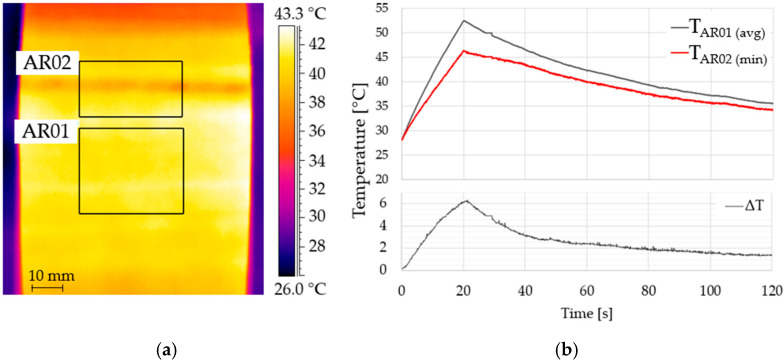
Results of the thermographic analysis of the composite polystyrene-filled profile: (**a**) surface temperature distribution at t = 10 s (heating); (**b**) temperature and thermal contrast versus time for reference and suspected locations. Note that the observed thermal pattern is influenced by the internal fill design and does not by itself confirm delamination; the quantitative geometric verification is provided by the 3D scan in Figure 9.

The analysis parameters for active infrared thermography were the same as for the earlier case. Figure 8 shows the temperature distribution at the tenth second of the analysis (heating stage), as well as the temperature characteristics in the highlighted areas (AR01 and AR02), plotted against time. Despite the notable differences in thermal characteristics seen in the graphs and thermogram, the anomaly detected by the damage detection system is due to the profile’s fill design. The system did not observe damage to the profile due to the lack of separation of the composite surface from the impact-induced infill. This proves that the use of infrared thermography as a stand-alone system for assessing the condition of the construction has clear limitations.

**Figure 9:**
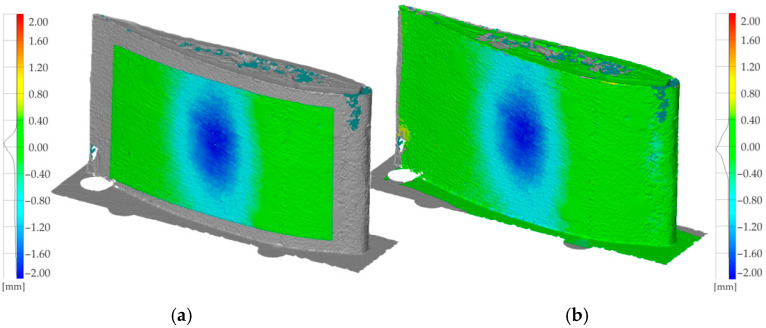
Quantitative surface-deviation analysis of the polystyrene-filled blade profile: (**a**) comparison to a polynomial reference surface; (**b**) comparison to a reference scan of an undamaged profile.

The thermogram and time-series metrics (temperature and thermal contrast) reflect the response of the surface to the thermal impulse; however, in the polystyrene-filled profile, the internal design can modulate heat-flow paths and yield thermal patterns that are not necessarily associated with delamination. Consequently, thermographic indications alone should be treated as qualitative and localization-oriented, pending quantitative geometric verification. We therefore performed a co-registered 3D scan to measure the surface deviation and confirm or refute the presence of a defect in the indicated region. Figure 9 presents the scan-based analysis, which consistently reveals a local indentation of approximately 2 mm, obtained both by comparison to a polynomial reference surface (Figure 9a), and to a reference scan of an undamaged profile (Figure 9b). This quantitative result aligns with the thermally anomalous area, thereby validating the indication from thermography and quantifying the magnitude of the defect, while minor technological gaps or features visible in the point cloud are not classified as defects by the comparative analysis.

The results clearly demonstrate the respective advantages and limitations of the two damage detection methods.

**Figure 10:**
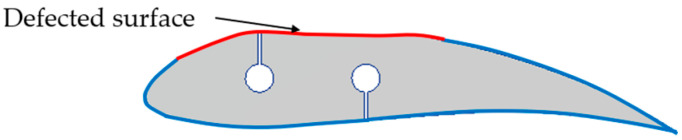
Schematic of the damage mechanism in the polystyrene-filled blade profile.

Active infrared thermography (IRT) reveals damage that perturbs heat flow, enabling qualitative detection and localisation of anomalies on thermograms. In the previously analysed empty GFRP profile (without fill) this thermal response was clear, while 3D scanning complemented the interpretation with geometric context. In the polystyrene-filled profile, the internal architecture modifies heat-flow paths, so a standalone thermographic inspection may either miss a surface indentation or mislead interpretations without prior knowledge of the internal design. Therefore, a co-registered 3D scan was employed to provide quantitative confirmation (e.g., measurement of ~2 mm indentation) and to distinguish true deformation from design-related thermal signatures. Figure 10 illustrates the damage configuration in the filled profile, which does not produce a pronounced disturbance of heat flow, explaining why an IR-only system may fail to detect it; in such cases, the scan determines both presence and magnitude. Consequently, combining IRT (qualitative detection and localisation) with 3D scanning (quantitative verification and measurement) increases diagnostic accuracy and reduces the probability of misinterpretation compared with either method in isolation.

### 3.2. Damage Detection in VAWT Wind Turbine Blade

To evaluate the coupled inspection workflow under unknown internal architecture, a 120 cm VAWT blade (polystyrene-filled with a glass-epoxy outer shell) was first digitised via 3D laser scanning and subsequently examined section-by-section using the active IRT setup described earlier. In this context, IRT with image-based region/point annotation provides a qualitative indication and localisation of potential anomalies on thermograms, whereas 3D scanning offers quantitative confirmation and measurement of geometric deviation. The test was performed without prior knowledge of the blade’s manufacturing history and without an available CAD model or reference scan.

**Figure 11:**
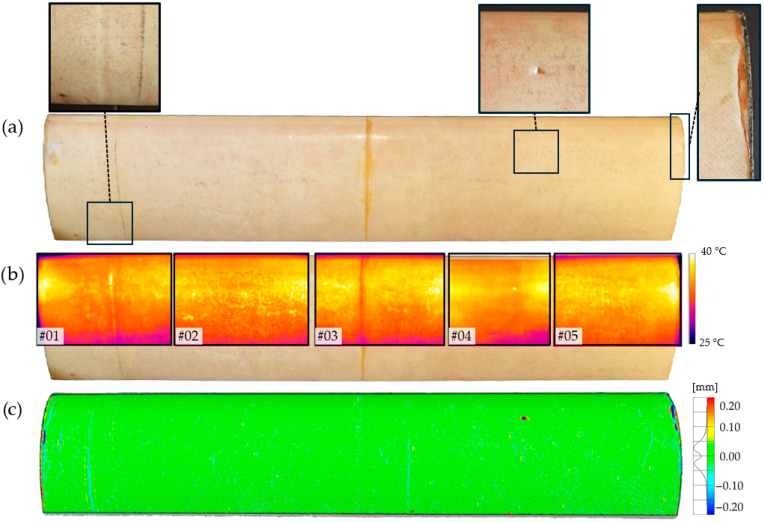
VAWT blade inspection with unknown internal architecture: (**a**) top view with five analysed areas (#1–#5); (**b**) thermograms recorded during heating (qualitative localisation); (**c**) object scan analysis based on comparison with a polynomial reference surface (quantitative verification).

Thermograms alone identify areas with altered heat-flow patterns but may conflate design-driven thermal signatures with damage-related ones when the internal structure is unknown. For the VAWT blade, the thermal responses in #1 and #3 coincide with bonding regions of the polystyrene fill, not with delamination; a subtle disturbance adjacent to the bonding line in #1 could be overlooked without a region-of-interest workflow. The impact-related indication in #4 and the edge disturbance on the right side require targeted confirmation. The co-registered scan-based deviation map in Figure 11c serves this role, discriminating true geometric change from internal architecture effects, thereby validating (or falsifying) the thermal indications.

**Figure 12:**
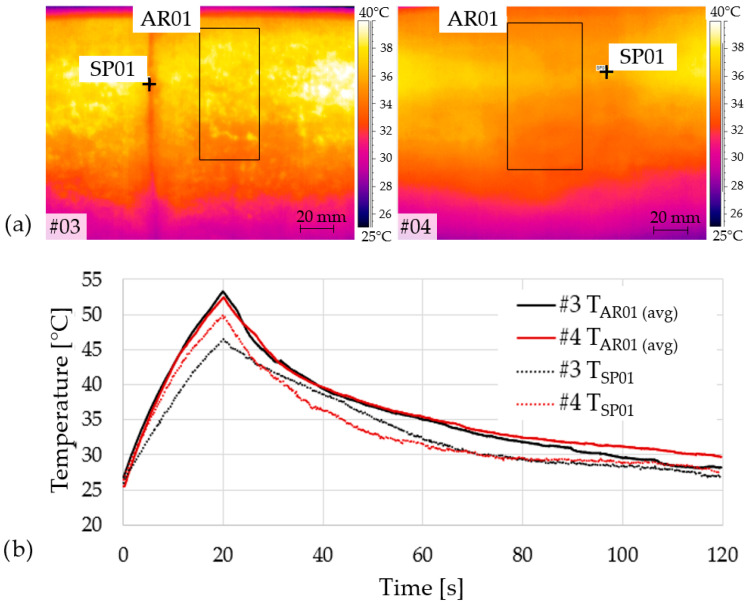
(**a**) Thermograms for areas #3 and #4 with AR01 (reference area) and SP01 (point at anomaly centre) indicated; (**b**) corresponding temperature–time curves and contrasts.

To quantify the thermal response, we analysed AR01 (reference area; average temperature) and SP01 (single point at the anomaly centre) for areas #3 and #4. During heating, T_SP01_ deviates markedly from T_AR01_, whereas in early cooling the point-wise signal converges faster, highlighting the practical advantage of area-based metrics and peak-contrast values for robust detection. Figure 12 illustrates thermograms with the annotated regions/points (a), and the corresponding temperature–time curves and contrasts (b).

Active infrared thermography supported by a scan analysis of the object allows for the unambiguous identification of surface damage that causes distortion to the surface of the test object. Using thermogram #3 and the scan analysis of this area as an example, it can be concluded that coupling the two damage detection methods correctly interprets the test results and avoids incorrectly indicating damage when the blade’s internal structure and design are responsible for its different thermal characteristics.

The VAWT blade inspection was performed without prior knowledge of the internal architecture and without an available CAD or reference scan, which imposes specific constraints on interpretation: (i) active IRT is sensitive to heating uniformity, emissivity, local geometry, and ROI selection; point-wise signals (e.g., SP01) may converge rapidly during cooling and are more noise-prone, hence area-based metrics and peak-contrast values during heating are preferred; (ii) internal fills and bonding features can mask or mimic thermal responses, generating false positives when using thermograms alone; (iii) 3D scanning accuracy depends on resolution, calibration and registration; absence of a CAD reference increases uncertainty and necessitates polynomial-surface comparisons; and (iv) outdoor conditions and large-curvature surfaces complicate co-registration between thermal imagery and 3D geometry, potentially introducing localisation error. Given these factors, the combined use of qualitative IRT (detection and localisation) with quantitative scan analysis (verification and measurement) is recommended to reduce both false positives (design-related thermal signatures) and false negatives (weak thermal contrast), improving diagnostic confidence for composite blades.

### 3.3. Damage Detection in Horizontal-Axis Wind Turbine (HAWT) Blades

A set of three horizontal-axis wind turbine (HAWT) blades was examined. Each blade had a length of 1.75 m and was protected by an outer weather-resistant layer (Figure 1a). Visual inspection revealed no significant surface damage. All blades were digitised at a nominal point spacing of 1 mm and inspected using local active infrared thermography (IRT) according to the test procedure described earlier. This workflow supports the study’s thesis: scan-based comparison provides global, quantitative geometry verification for fleet homogeneity, whereas IRT delivers local, qualitative anomaly localisation on thermograms.

**Figure 13:**
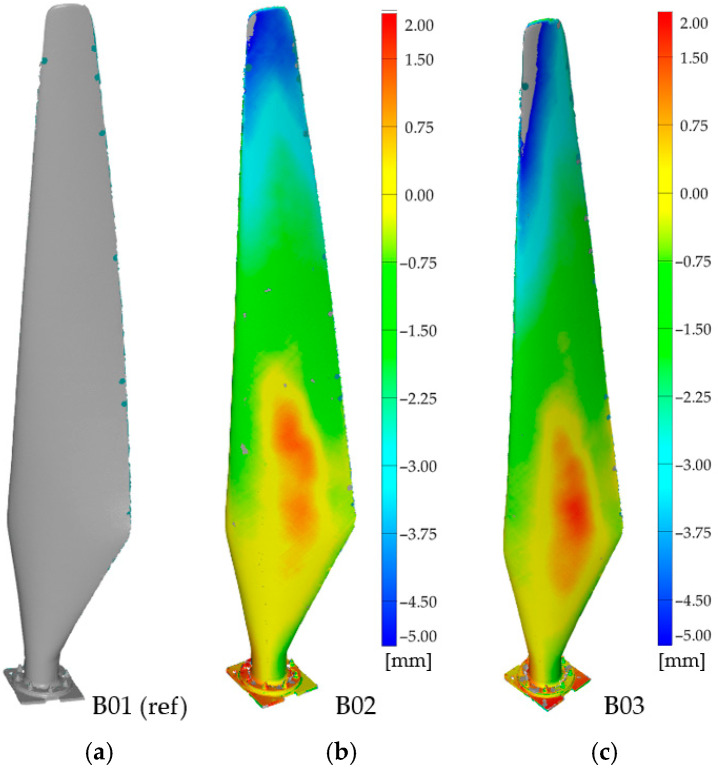
Comparative analysis results indicating geometric deviation between blades (**b**) B02 and (**c**) B03 relative to (**a**) B01 (reference in this stage).

For multi-blade sets, consistency of mass and geometry is essential; deviations can lead to unbalanced operation and elevated risk. We therefore adopted a comparative workflow in which one blade scan serves as a temporary reference, while the remaining scans are compared against it. In the initial stage, B01 was used as the reference and B02/B03 were compared against it. The resulting deviation maps showed no local damage, but indicated a systematic geometry difference for B02 and B03 relative to B01, with a tip-region deviation of approximately 5 mm over the 1.75 m length (Figure 13). This highlights the importance of documenting the chosen reference when no ideal CAD baseline is available.

**Figure 14:**
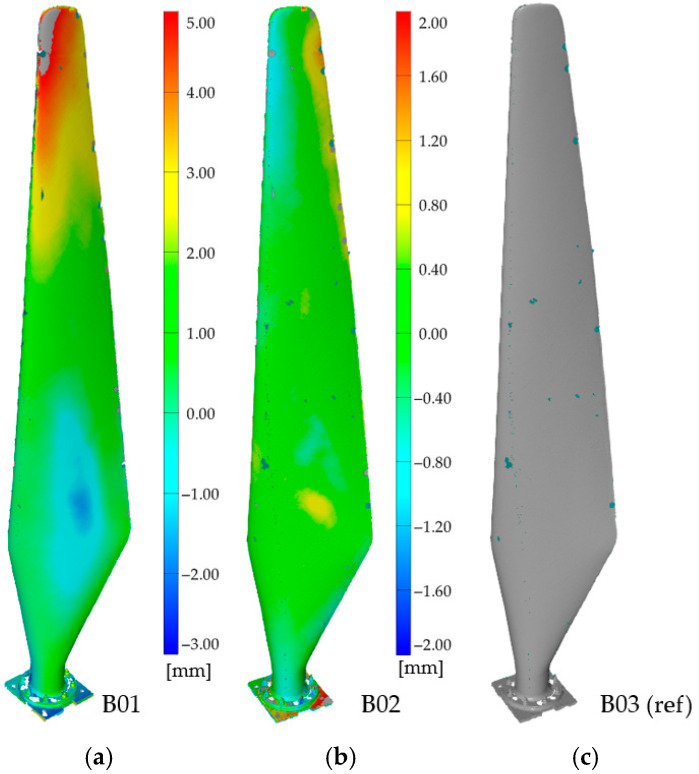
Comparative analysis results indicating geometric deviation between blades (**a**) B01 and (**b**) B02 relative to (**c**) B03 (reference in this stage).

To further assess the impact of reference choice, B03 was next used as the reference. In this configuration, B02 exhibited a nearly uniform deviation close to 0 mm, with no visible local damage, while B01 showed a larger deviation, particularly near the tip—demonstrating that B01 differs in quality from the other two blades (Figure 14). This confirms that, without a design-baseline model, reference selection must be interpreted carefully to avoid superficial conclusions about blade quality.

**Figure 15:**
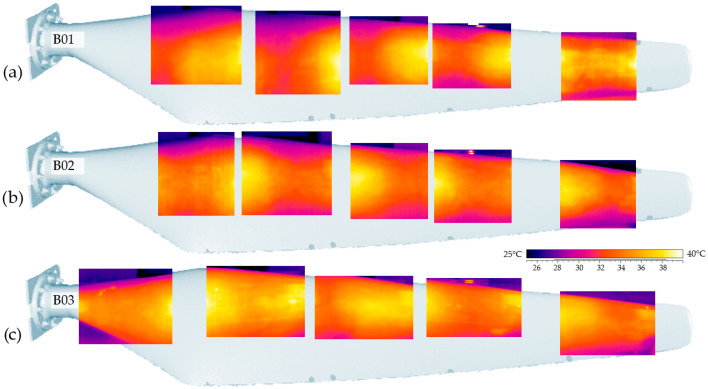
Results of a local active infrared thermography survey for selected areas of the wing: (**a**) B01, (**b**) B02, (**c**) B03.

Local active IRT was then applied to selected regions on B01–B03. Because of resolution constraints of thermal cameras at large stand-off distances and the challenge of uniformly heating complex curved surfaces, full-surface IRT would reduce temperature-field fidelity and may miss subtle variations. Consequently, in large, geometrically complex blades, stand-alone IRT is best suited for the local verification of selected areas, whereas scan-based comparison provides global homogeneity checks and quantitative thresholds for acceptance. This division of roles improves the interpretability and reliability of the overall non-destructive evaluation (see Figure 15 for representative local IRT panels).

The IRT survey did not reveal clear local changes indicating structural damage. Minor thermal anomalies visible on B03 corresponded to small surface features and therefore did not affect the overall quality assessment. In line with the study’s thesis and prior sections, combining methods increases diagnostic confidence: IRT provides qualitative localisation (detecting where an anomaly may occur), while scan analysis delivers quantitative verification (measuring whether—and by how much—geometry deviates). Used together, these methods reduce the chance of false positives (design-related thermal signatures) and false negatives (weak thermal contrast), thus improving the accuracy and robustness of quality control for composite HAWT blades.

## 4. Discussion

Early stage structural condition monitoring aims to identify changes that, if left undetected, could lead to significant degradation or failure under normal operating conditions. In the context of wind turbine components, modern non-destructive testing (NDT) workflows have a dual role: (i) detecting and localising potential damage, and (ii) enabling informed decisions on maintenance, safe life extension, and periodic inspections targeted at previously recorded anomalies. The results presented across multiple scales of wind turbine structures show that using a single method in isolation (quality control, condition monitoring, or damage detection) can bias interpretation—either by under-detecting meaningful indications, or by over-interpreting design-related features. In contrast, combining complementary techniques leverages the strengths of each modality while mitigating their limitations.

For composite profiles with polystyrene fill and for surface-only damage (without clear separation between the laminate and the core), active infrared thermography (IRT) may not reflect the actual condition of the surface, because internal features and fill architecture modulate heat-flow paths and attenuate thermal contrast. In such situations, reverse-engineering (RE) scan analysis provides quantitative geometry deviation (e.g., local indentation), allowing a cross-check of thermographic indications and a disambiguation between genuine defects and design-induced patterns. The VAWT blade case study confirms that thermography alone may yield false indications linked to manufacturing details or the internal layout; integrating 3D scanning clarifies the presence and magnitude of geometric change and stabilises the overall assessment of structural condition.

For HAWT blades (fleet comparison), the scan-based workflow enables homogeneity checks under the absence of an ideal CAD baseline: choosing a temporary reference (e.g., B01 or B03) and comparing the remaining blades produces consistent deviation maps indicating which blade differs in overall geometry (e.g., ~5 mm tip-region offset over 1.75 m), even when local IRT does not reveal any clear thermal anomalies at the measurement resolution. Practically, large curved geometries pose two challenges for IRT: (i) resolution and stand-off distance, and (ii) uniform heating. As a result, stand-alone IRT is best suited for local verification, while 3D scan comparison provides fleet-level quantitative thresholds and reference-aware judgement. Together, these observations support the central premise: IRT (qualitative localisation) combined with scanning (quantitative verification) improves diagnostic confidence and reduces both false positives (design-related thermal signatures) and false negatives (weak thermal contrast).

## 5. Conclusions

The study evaluated active infrared thermography and laser-based 3D scanning for quality assessment and damage detection in wind turbine structures. The main conclusions are:

- Active infrared thermography effectively detects surface-level damage where thermal continuity is broken (e.g., cracks, delamination) and provides spatial localisation on thermograms. Its effectiveness decreases for minor geometric changes (e.g., shallow dents) and when internal elements of the blade (fills, bonding features) mask the thermal response of the inspected area.
- Reverse-engineering techniques (surface mapping, comparisons to polynomial references or available scans) detect small geometric deviations and quantify their magnitude. While 3D scanning excels at external geometry change, it is not intended to directly reveal internal structural changes unless they manifest at the surface or in measurable dimensional deviations.
- Using IRT or scanning independently can introduce method-specific biases: IRT is sensitive to heating uniformity, emissivity, local geometry, and ROI selection; scanning depends on resolution, calibration, registration, and the availability of a reference. Consequently, conclusions based on a single modality may be incomplete or misleading.
- A coupled IRT-and-scan workflow reduces technological limitations and enables cross-verification of results, increasing accuracy, robustness, and confidence in structural condition assessment and quality control across scales (specimens, VAWT, HAWT).

Rapid advances in compact, high-accuracy laser scanners (point rate, precision) and the proliferation of UAV platforms make coupled quality control systems increasingly practical for hard-to-access structures. A key next step is to validate passive infrared thermography (without an active heat pulse) alongside laser scanning for UAV-enabled inspection campaigns under real environmental conditions (temperature, solar irradiance, wind). Passive monitoring identifies areas of thermal contrast relative to surroundings; integrating it with on-board scanning could provide a direct, geometry-aware inspection route for large composite blades without the need for external heating.

## Data Availability

The original contributions presented in this study are included in the article. Further inquiries can be directed to the corresponding author.
